# Sudden Unexplained Nocturnal Death Syndrome in Central China (Hubei): A 16-Year Retrospective Study of Autopsy Cases

**DOI:** 10.1097/MD.0000000000002882

**Published:** 2016-03-07

**Authors:** Zhenglian Chen, Jiao Mu, Xinshan Chen, Hongmei Dong

**Affiliations:** From the Department of Forensic Medicine, Tongji Medical College, Huazhong University of Science and Technology, Wuhan (ZC, XC, HD), and Department of Pathology, HeBei North University, Zhangjiakou, Hebei (JM), P.R. China.

## Abstract

A retrospective study was conducted at Tongji Forensic Medical Center in Hubei (TFMCH) from 1999 to 2014. Forty-nine cases of sudden unexplained nocturnal death syndrome (SUNDS) were collected. The SUNDS rate was 1.0% in the total number of cases, in which an incidence was fluctuating over the years. Interestingly, April and January, and 3:00 to 6:00 am were the peak months and times of death. Among the decedents, farmers and migrant workers accounted for 67.3%. The syndrome predominantly attacked males in their 30s. One victim had sinus tachycardia. Thirteen victims (26.5%) were witnessed and had abnormal symptoms near death. Macroscopically, compared to sudden noncardiac deaths, the weights of brain, heart, and lungs had no statistical difference in SUNDS. Microscopically, the incidence of lung edema (45 cases, 91.8%) was significantly higher in SUNDS group than in the control group (27 cases, 55.1%). 82.9% of 35 SUNDS cases examined displayed minor histological anomalies of the cardiac conduction system (CCS), including mild or moderate fatty, fibrous or fibrofatty tissue replacement, insignificant stenosis of node artery, and punctate hemorrhage in the node area. These findings suggested that minor CCS abnormalities might be the substrates for some SUNDS deaths. Therefore, SUNDS victims might suffer ventricular fibrillation and acute cardiopulmonary failure before death. Further in-depth studies are needed to unveil the underlying mechanisms of SUNDS.

## INTRODUCTION

Sudden unexplained nocturnal death syndrome (SUNDS) has gained attention all over the world since it was first reported in 1917.^1^ The syndrome is featured by predominance of healthy young males without significant medical history, who suddenly die during sleep.^2^ The disease is termed “Bangungut” in Philippines,^1^ “Lai Tai” in Thailand,^2^ “Pokkuri Death Syndrome” in Japan,^3^ “Dream Disease” in Hawaii, “Sudden manhood death syndrome” in mainland China,^4^ and “sudden adult death syndrome” in England.^5^

SUNDS occurs frequently in Southeast Asia. In China, SUNDS mainly happens in the South. SUNDS prevalence is estimated at 38 per 100,000 men aged 20 to 49 years in Thailand,^6^ 43 per 100,000 individuals each year in Philippines,^7^ and 1/100,000 persons per year in southern China.^4^ The mechanisms of death due to SUNDS remain unclear, although malfunction of ion channel,^8^ nocturnal hypoxia, toxigenic bacteria,^9^ coronary arterial spasm,^3^ ventricular fibrillation, sleep apnea,^10^ and low potassium status^11^ are suspected.

Here, we report a 16-year retrospective study of 49 SUNDS cases conducted at Tongji Forensic Medical Center in Hubei (TFMCH). TFMCH is located in Wuhan, located in the middle of China. It is one of the first forensic institutions open to the public, including police offices, public health departments, hospitals, and so on. Because of its specific location, most cases in TFMCH are distributed in Hubei and surrounding provinces such as Henan and Hunan. Therefore, the incidence of SUNDS found in TFMCH could reflect its morbidity in central China. This study aimed to assess the epidemiology characteristics of SUNDS in central China and observe its underlying pathological changes.

## METHODS

### Ethical Statements

The research protocols were approved by the ethics committee of Huazhong University of Science and Technology. Informed consent was obtained from each claimed case.

### Study Design and Participants

The total cases in TFMCH were reviewed from January 1999 to December 2014. The cause of death for each victim was identified according to autopsy finding, histological examination, toxicology analysis, and investigation report (including clinical history and witness testimony). Name, gender, age, occupation, time of death, symptoms before sleep and near death, family history, as well as macroscopic and microscopic examinations of vital organs were reviewed.

Inclusion criteria of this survey were sudden, nonviolent death within 24 hours; seemingly healthy individual dying during sleep; no acknowledged lethal anatomical or histological changes; negative toxicological evaluation; age between 15 and 55 (inclusive). Excluded were cases with severe tissue decomposition; lethal diseases or pathological changes; and severe cardiac morphological lesions, like cardiac hypertrophy and serious cardiac conduction system (CCS) abnormalies.^4,12^

Totally 49 cases were diagnosed as SUNDS. During the same period, 49 cases of sudden noncardiac deaths, that matched the SUNDS by age and gender, were selected as control group. The control group included 31 sudden death cases died of severe craniocerebral injury, 10 cases of mechanical asphyxia and 8 cases of electrothanasia.

### Macroscopic and Microscopic Examinations

Vital organ weights and ventricular wall thickness were measured after fixation in formalin. All organs were assessed by routine histology. Two subgroups were established and included according to victims above and below 19, respectively when the weight of organs was observed. Among all the selected cases, the CCSs were examined in 35 SUNDS deaths and 30 control cases using the method of Song et al.^13^ Based on Song et al's study,^13,14^ the adipose or fibrous tissue in CCS was increased with age, so it was vital to divided the lesions into mild (Grade I, <25%), moderate (Grade II, 25–50%), and severe degrees (Grade III, >50%). In the present study, we excluded serious CCS anomalies, which included moderate or severe fatty or fibrosis infiltration of sinoatrial node (SAN) and His bundle (HB) below 40 and severe fatty or fibrosis replacement above 40 and below 60 years old, moderate or severe atrioventricular node (AVN) fatty infiltration below 30 and severe infiltration above 30 and below 50 years old, massive or focal hemorrhage around the mode, and stenosis of node artery >75%.^15^

### Statistical Analysis

Statistical analyses were conducted by SPSS 20.0. Continuous data are presented as mean ± standard deviation, whereas categorical variables were expressed as number and/or percentage. Independent samples *t* test was performed for continuous data. Chi-square test for 2 × 2 table was used to compare the significance for categorical data. A 2-tailed *P*-value < 0.05 was considered statistically significant.

## RESULTS

### Incidence by Year and Month, Time and Place of Death

**FIGURE 1:**
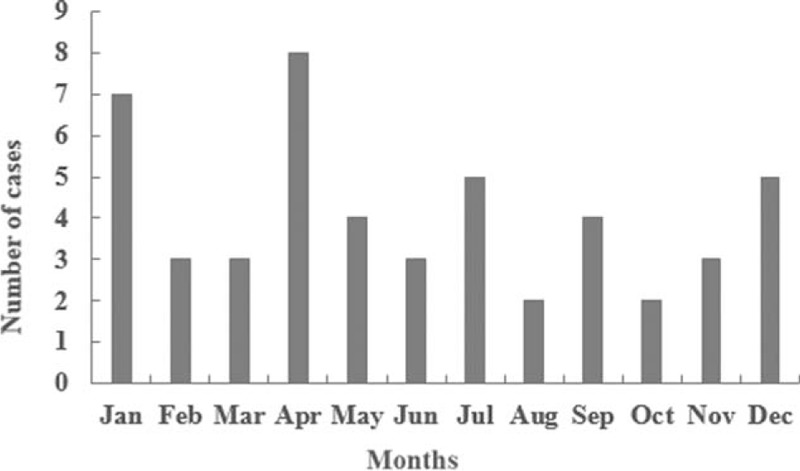
Seasonal incidence of SUNDS cases.

The total cases examined in our center were 5106 from January 1999 to December 2014. The yearly rates of SUNDS cases ranged from 0.3% to 2.2%, averaging 1.0% and in a fluctuating trend. The occurrence peaked in April (8 cases, 16.3%), followed by January (7 cases, 14.3%) (Figure 1).

Death mainly occurred between 3:00 and 6:00 am during the night. Among the 49 cases, 9 victims (18.4%) received hospital care, 7 (14.3%) died in the emergency room, and 2 (4.1%) died in the ambulance. The remaining 40 cases (81.6%) were found dead in bed.

### Age and Gender Distribution

**FIGURE 2:**
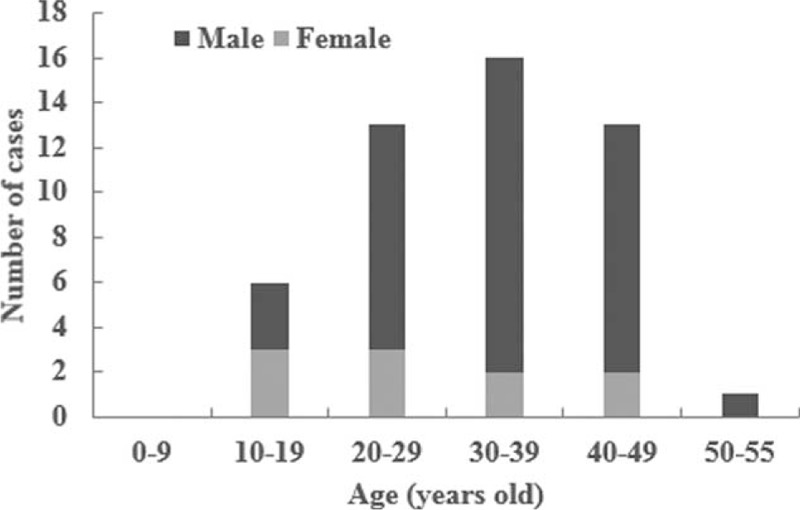
Incidence of SUNDS by age.

The age at death were 32.6 ± 10.3 years, with the incidence peaking between 30 and 39 years (16 cases, 32.7%). The least affected age group was 50 to 59 years (1 case, 2.0%) (Figure 2).

Of the 49 SUNDS cases, 10 were females at the age of 26.7 ± 9.5 years, ranging from 16 to 40 years; the remaining 39 cases were males of 34.1 ± 10.0 (18–55) years. The ratio of male to female was 3.9.

### Education Background and Vocation Distribution

One male had a PHD degree, and the remaining victims had different levels of education, including middle- or high-school experience, even illiterate background. Farmers accounted for 44.9% of the decedents (22 cases); 11 (22.4%) were migrate workers, including 3 construction worker, 2 print workers, 2 traders, 1 barber, 1 carpenter, 1 bartender, and 1 coke plant worker; there were 8 (16.3%) prisoners, 4 (8.2%) students (1 PhD and 3 high school students), and 4 (8.2%) public servants (1 policewoman, 2 soldiers, and 1 railway staff) affected.

### The Situations Before Sleep and at Near Death

Of all cases, the situations before sleep in 10 cases (20.4%) were known, and can be grouped into 3 main categories: big meal and small amount of alcohol; bad mood; abnormal symptoms and signs: tachypnea, tachycardia, left chest pain with hand paralysis, headache, fever, and seizure-like symptoms (foaming at mouth and tetany). The situations were unclear for the remaining victims.

Of the 49 cases, 13 were witnessed (26.5%) and had acute symptoms or signs at near-death time: abnormal snoring, seizure-like activities, screaming, clutching to people around, and tachycardia.

### Family History and Abnormal ECG in Clinical History

Two victims (4.1%) had family history of sudden unexplained death of first-degree relatives. One boy's mother and elder sister died in sleep with unknown causes. The other male victim's 2 elder brothers died in sleep. But the dead relatives of the 2 cases were not performed autopsy.

One case (2.0%) had abnormal electrocardiogram (ECG) of sinus tachycardia in clinical history, with ventricular rate of 140 beats/min.

### Macroscopic Findings

As are shown in Tables 1 and 2, the brain, heart, and lungs weights of SUNDS were comparable to the controls (brain: 1449.2 ± 120.2 vs 1444.8 ± 183.6 g, *P* > 0.05; heart: 310.4 ± 48.4 vs 304.8 ± 60.0 g, *P* > 0.05; lungs: 1039.9 ± 296.7 vs 1017.5 ± 350.7 g, *P* > 0.05). The left or right ventricular thickness had no statistically significant difference between SUNDS and controls (left ventricular: 12.0 ± 1.4 vs 11.8 ± 1.9 mm, *P* > 0.05; right ventricular: 3.1 ± 0.8 vs 3.2 ± 1.0 mm, *P* > 0.05).

**TABLE 1:**
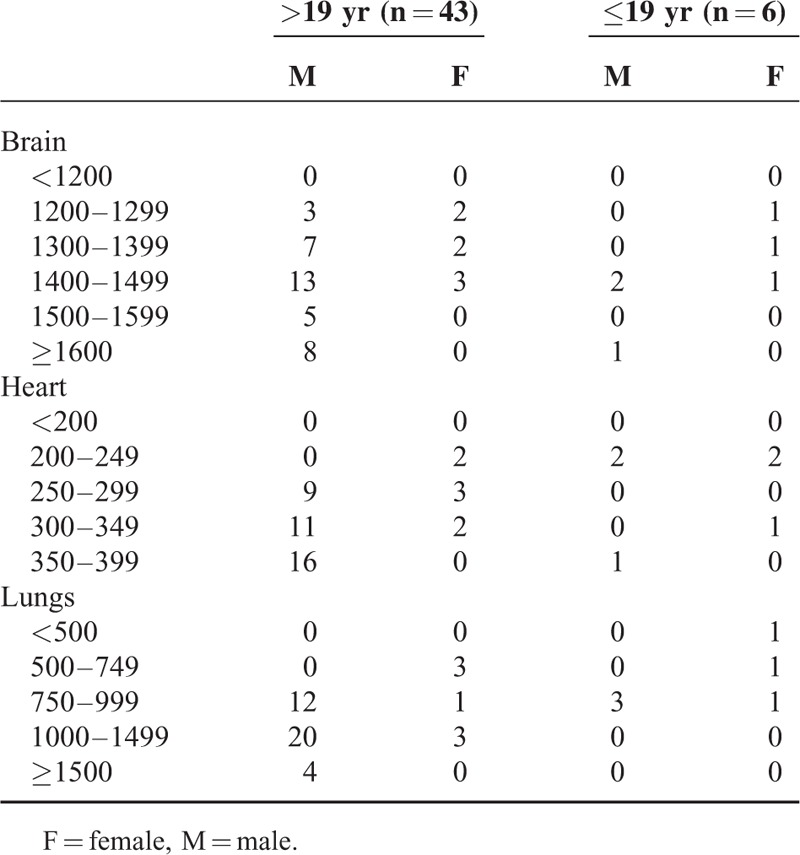
Vital Organ Weights of SUNDS Cases by Gender and Age

**TABLE 2:**
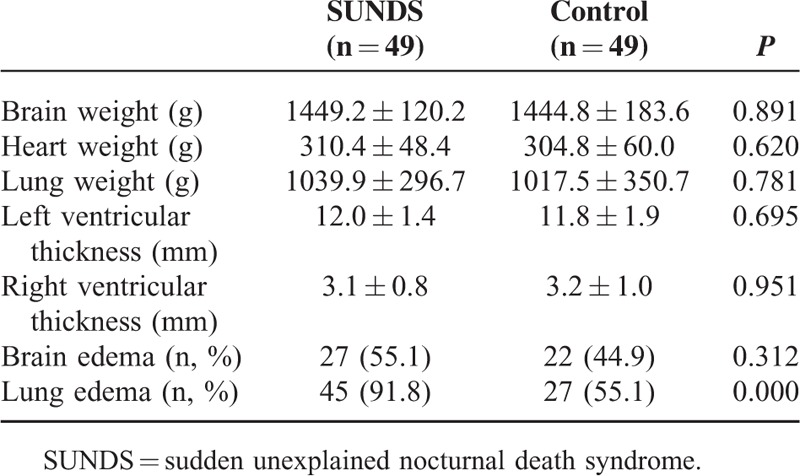
Macroscopic and Histological Analysis of Vital Organs Compared to the Control Group (Except the Cardiac Conduction System)

### Microscopic Findings

The CCSs in 35 SUNDS cases was examined and showed abnormalities in 29 victims. Solitary SAN abnormalities were observed in 2 cases (5.7%), while solitary AVN or HB anomalies were found in 9 cases (25.7%) or 2 cases (5.7%), respectively; both SAN and AVN abnormalities were observed in 13 cases (37.1%), and simultaneous SAN, AVN, and HB anomalies were found in 3 cases (8.6%). From a different perspective, minor fatty infiltration in SAN/AVN/HB was observed in 22 (62.9%), fibrosis in 12 (34.3%), punctate hemorrhage in the node area in 5 (14.3%), fibrofatty infiltration in 3 (8.6%), and minor stenosis of node artery in 2 (5.7%) cases (Table 3).

**TABLE 3:**
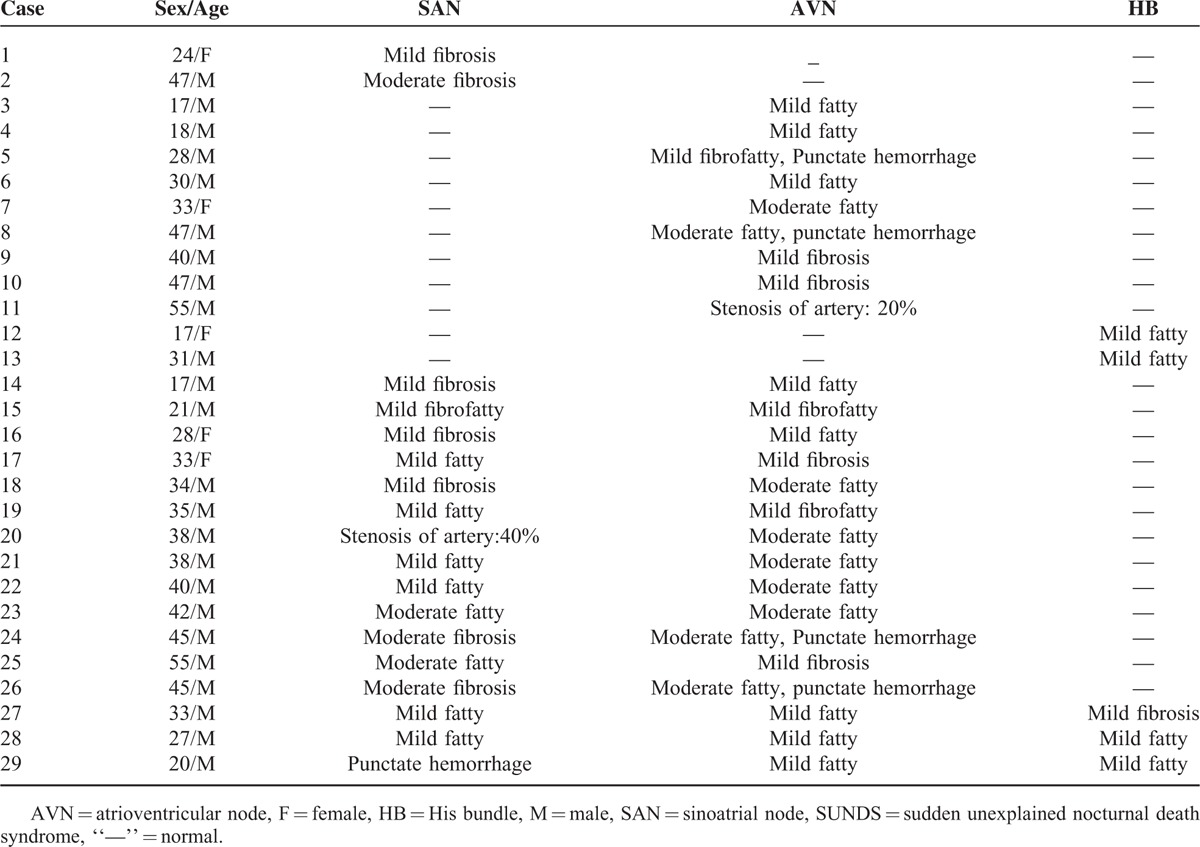
Summary of Abnormal Cardiac Conduction Systems in SUNDS

The incidence of SAN (18 cases, 51.4%) and AVN abnormalities (25 cases, 71.4%) was higher in SUNDS victims than that in the control group (3 cases, 10% and 6 cases, 20%, *P* < 0.001). However, the abnormalities of HB was not statistically different (5 cases, 14.3% vs 5 cases, 16.7%; *P* > 0.05) (Table 4).

**TABLE 4:**
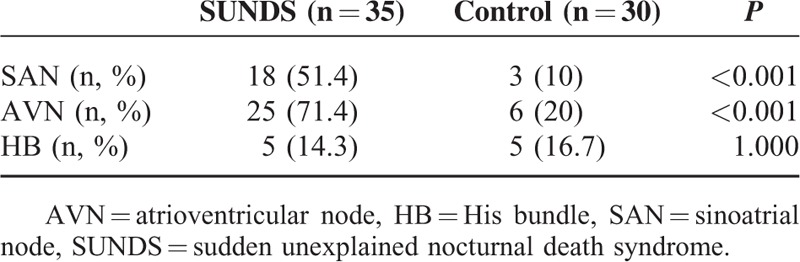
The Abnormal Cardiac Conduction System Analysis of SUNDS

Besides the above-mentioned lesions, cerebral edema was observed in 27 cases (55.1%) of SUNDS group, which had no difference from the control group (22 cases, 44.9%, *P* > 0.05). However, pulmonary edema was higher in SUNDS victims (45 cases, 91.8%) than in the controls (27 cases, 55.1%, *P* < 0.001) (Table 2). No other specific pathologies were noted in other internal organs except congestion.

As for the one presented with sinus tachycardia in the clinical history, mild fibrous tissue replacement in SAN was observed.

## DISCUSSION

Although SUNDS was acknowledged for many years, it was still completely inconsistent in many aspects, even about its nomenclature and diagnosis criteria. The inclusion criteria of SUNDS were broad a few decades ago, and therefore the incidence of SUNDS might be expanded. However, the criteria have been developed in the recent years. The obvious morphological and histological abnormalities of the heart which have been recognized as SUNDS, such as cardiomyopathy, cardiac hypertrophy, and serious CCS disorders, were excluded from SUNDS cases.^16^

Searched SUNDS from Pubmed, most literatures were about Brugada syndrome (BrS). BrS was characterized by right bundle branch block, ST-segment elevation in leads V1, V2, and V3, and an increased risk of SCD due to ventricular fibrillation.^17^ Clinically, patients of BrS had nocturnal agonal respiration, showed male predominance, usually died at sleep or at rest, and had a high risk of ventricular tachycardia/fibrillation, which were also reported in SUNDS victims.^18,19^ Genetically, SUNDS and BrS shared cardiac sodium channel alpha submit mutations, which encoded the cardiac sodium channel.^20,21^ In the autopsy studies, Morimoto et al^22^ found in a 30-year-old man with BrS, who died suddenly, had fibrofatty replacement of SAN. Similarly, we found minor anatomic conduction system abnormalities in 82.9% of examined SUNDS deaths. All the current and previous studies implicated that SUNDS and BrS were very likely the allelic disease.

Munger and Booton^23^ concluded a marked decrease in SUNDS morbidity after World War II in Japan and for Southeast Asia refugees after they have lived for a long time in America, indicating that lifestyle development might contribute to its morbidity. The yearly trend in China has not yet been reported. In our investigation, the SUNDS rate was approximately 0.3% to 2.2% annually in TFMCH, and showed a fluctuating trend year-by-year. Although living standard has greatly been developed, SUNDS still is a threat on the health of people.

SUNDS happens in seemingly healthy male adults. Indeed, we found that males were 3.9 times more affected than females. Most decedents were less-educated farmers and migrant workers in their 30s. Interestingly, Wong et al^24^ reported that Thai construction workers in Singapore show high SUNDS occurrence rates. In addition, Tungsanga and Sriboonlue^6^ found that about 75% of SUNDS victims have a low annual income. These may be attributed to the following reasons: the adult males were confronted with higher psychological and physical stresses.^4^ Furthermore, as important risk indicator of SUNDS, cardiac autonomic neuropathy was more common in males.^25^ Thus, SUNDS was more prone to be males; especially manual workers often have high levels of fatigue. In addition, some victims suffered from stresses like bad mood before death. Therefore, more attention should be paid to psychological and physical factors in SUNDS.

The reported peak months for SUNDS are generally in summer.^26^ Southeast Asia and South of China have a tropical monsoon climate, characterized by high temperatures all year round. The high-occurrence seasons in these places have higher temperatures with high humidity. The infection was suspected to be related with the high incidence of SUNDS in these regions. Indeed, Yap et al^27^ discovered that Thai in Singapore suffered from SUNDS showed high occurrence of melioidosis bacterial infection. In addition, pyrogenic toxins of *Staphylococcus aureus* have been identified in 2 cases of sudden unexpected death in adults in UK, which suggested that these or other toxins might induce inflammatory responses resulting in SUNDS.^9^ We found that most deaths happened in April and January, when the climate suddenly turns hotter or coldest in central China around Hubei. The uncomfortable climatic conditions are at a high risk of cold and infection. The antemortem symptoms such as headache and fever in our study support cold and infection as possible risk factors of SUNDS. The relationship between seasons and infection in SUNDS deserves further exploration.

It was believed that oscillations in the automatic nervous activity might be essential to SUNDS. Most SUNDS deaths occurred in the sleep at night in our study. Wichter^28^ showed that ventricular arrhythmias and even SCD occurred at rest or during sleep when the vagal modulation was dominant. Moreover, sleep-disordered breathing could elicit marked autonomic changes during sleep. Ikeda et al^29^ found that high vagal tone predominated when people have a big meal before sleep. The symptoms of the decedents before sleep and at near death also support the hypothesis that the role of the automatic nervous activity in SUNDS, but it needs to be further explored.

CCS generates and propagates action potentials that lead to atrial contraction and subsequent ventricular contraction. In the network, SAN gives rise to the initial depolarization, then intraatrial conduction pathway myocytes carry the impulse into AVN. HB provides an efficient way by which the electrical signal received from AVN is delivered to left and right bundle branches for distribution to ventricular myocardium. Several researchers documented the correlation between CCS pathological abnormalities and arrhythmias.^30–32^ If the parenchymal cells of CCS were replaced by fibrosis and fatty tissue, these changes led to the disconnection between the node cell, the node, and periphery, even the abruption of the impulses. Eventually bradycardia, arterial or ventricular fibrillation, and sudden death happened.^14^ Csepe et al^33^ reported that pathological fibrosis upregulation within the SAN might lead to tachycardia–bradycardia arrhythmias and cardiac arrest due to SAN reentry and exit block. Song et al^34^ reported that SAN fatty infiltration could lead to bradycardia. Serious CCS anomalies could lead to SCD, therefore they were excluded in the present study. The abnormalities of CCS have also been described in SUNDS but remained controversial. Okada and Kawai^35^ have demonstrated that SAN fibrosis with slight anomalies of the SAN artery was specifically seen in the sudden death of apparently healthy young males. Some researchers believed the abnormalities of CCS might be substrates for SUNDS.^36,37^ However, in a recent study reported by Gervacio et al,^38^ the CCSs of 5 examined SUNDS cases were normal. In our study, 82.9% of the examined cases had minor CCS changes mild or moderate fatty, fibrous or fibrofatty tissue replacement, insignificant stenosis of node artery, and punctate hemorrhage in the node area. Moreover, mild SAN fibrosis was observed in one victim with tachycardia in clinical history. Matturri et al^39^ found that CCS hemorrhage was also seen in cases of sudden infant death syndrome. In this study, tachycardia before sleep and near death was found in some victims. Thus, it is implied that anomalous CCSs, which are related to arrhythmia, may contribute to some SUNDS deaths.

Recently, the coronary arteries were showed to be narrow in SUNDS. The narrowed coronary arteries might be associated with occurrence of severe coronary arteries spasm. It was inferred that SUNDS might be a syndrome caused by severe spasm of coronary arteries occurred under certain conditions, such as postprandial remnant hyperlipoproteinemia.^3^ The relation between SUNDS and coronary artery stenosis and atherosclerosis need to be studied in the future research.

The autopsy findings were reviewed in this study. The weights of the brain, heart, and lung were not statistically different between 2 groups. The brain edema was observed in 55.1% of the cases, although there was no difference compared to the control group. It was well known that the brain edema was associated with cerebral injury, hypoxia, and ischemia. The plausible explanation about the brain edema of this study was that the victims underwent a hypoxemia status before death. Abnormal snoring in the witnessed cases of this study indicated that the decedents might have suffered obstructive sleep apnea and subsequent hypoxia. The brain edema might have in turn increased the obstructive sleep apnea. It was known that obstructive sleep apnea and low oxygen increased the incidence of related arrhythmia.^40^

Generally, lung edema was caused by cardiogenic or noncardiogenic causes. The cardiogenic condition was mainly because of left ventricular failure, severe arrhythmias, severe heart attack, hypertensive crisis, and fluid overload. In our study, the incidence of pulmonary edema in SUNDS victims was statistically higher than the controls. However, the lung weight of SUNDS had no statistical difference from the controls, although a mildly increasing tendency in SUNDS was observed. The reason for the disparity of the lung weight and pulmonary edema was not clear. Possible explanations was that lung weight not only depended on the incidence, degrees, and ranges of pulmonary edema, but also were associated with the lung volume, the cause of death, survival time, the severity and duration of heart failure, gender, age, and etc.^41–43^ Combined with the symptoms before death in SUNDS victims and heart examination, we inferred that the SUNDS decedents before death might have suffered acute cardiopulmonary failure.

In conclusion, we speculated that minor CCS abnormalities might be anatomic basis for SUNDS, which contributed to subsequent arrhythmia. Acute cardiopulmonary failure occurred under a certain conditions, such as climate abnormality, stress, and even influenza. These findings in the present study have potential implications for SUNDS mechanisms and pathogenesis.

## CONCLUSION

This is the first study assessing the epidemiological characteristics and possible pathological changes of SUNDS in central China. Our findings indicated that minor abnormalities of CCS might be related to SUNDS deaths. In routine postmortem examinations, a detailed study of the CCS should be emphasized in ambiguous sudden deaths, although the histological examination of CCS is tedious. Furthermore, a combination of medicolegal and molecular autopsies is needed to explore the possible etiology of SUNDS in the future.
